# 

**Published:** 1909-02-13

**Authors:** 


### GRADUATES' MEDICAL SCHOOLS.

DIARY FOR WEEK FEB. 15 TO FEB. 20.

**NORTH-EAST LONDON POST-GRADUATE COLLEGE, Prince of Wales's General Hospital, Tottenham, N.**

**At 4.30 p.m.**
**Feb. 16,** Mr. J. H. Evans, **Tumours of the Female Breast.**
**Feb. 18,** Mr. W. Edmunds, **The Diagnosis and Treatment of Fractures.**

**LONDON SCHOOL OF CLINICAL MEDICINE, Seamen's Hospital, Greenwich, S.E.**

**At 3.15 p.m.**
**Feb. 15,** Mr. Turner, **Some Common Rectal Complaints.**
**At 2.15 p.m.**
**Feb. 16,** Dr. Russell Wells, **Aortic Disease.**

**NATIONAL HOSPITAL FOR THE PARALYSED AND EPILEPTIC, Queen Sq., Bloomsbury, W.C.**

**At 3.30 p.m.**
**Feb. 16,** Sir Victor Horsley, **Surgery of the Nervous System.**
**Feb. 19,** Dr. Collier, **The Treatment of Sciatica and Allied Affections.**

**THE THROAT HOSPITAL, Golden Square, W.**

**At 5.30 p.m.**
**Feb. 15,** Mr. Faulder, **Neuroses and New Growths of the Pharynx,**
**Feb. 18,** Mr. Faulder, **Injuries and Foreign Bodies of the Pharynx and Larynx.**

**ST. JOHN'S HOSPITAL FOR DISEASES OF THE SKIN, Leicester Square, W.C.**

**Feb. 18,** Chesterfield Lectures, **Syphilis as it Modifies Other Eruptions.**

**THE POST-GRADUATE COLLEGE, West London Hospital, Hammersmith, W.**

**At 10 a.m.**
**Feb. 15 and 18,** Surgical Registrar, **Demonstration.**
**Feb. 19,** Medical Registrar, **Demonstration.**
**At 12 noon.**
**Feb. 15,** Dr. Low, **Pathological Demonstration.**
**At 12.15 p.m.**
**Feb. 17 and 20,** Dr. Pritchard, **Practical Medicine.**
**At 5 p.m.**
**Feb. 15,** Dr. Low, **Sleeping Sickness.**
**Feb. 16,** Dr. Robinson, **Gynæcology.**
**Feb. 17,** Mr. Baldwin, **Surgery.**
**Feb. 18,** Mr. Keetley, **Clinical.**
**Feb. 19,** Dr. Hood, **Some Clinical Observations on Heart Disease.**

### FORTHCOMING DINNERS.

The anniversary dinner of the Medical Society of London will be held at the Whitehall Rooms on Wednesday, March 10, at 7.30 p.m.

The annual dinner of the Chelsea Clinical Society will take place at the Gaiety Restaurant, Strand, W.C., on Thursday, March 4. Particulars can be obtained from Dr. Collis Hallowes, 104 Buckingham Palace Road, S.W.

Lord Robert Cecil, M.P., will preside at a dinner of the staff and students of the London School of Clinical Medicine (Post-Graduate) at the Savoy Hotel on Friday, February 19. Among those who will attend are Sir T. Barlow, Sir H. Burdett, Sir W. Bennett, Sir R. Burnet, Surgeon-General A. M. Branfoot, Sir W. J. Collins (Vice-Chancellor of the University of London), Sir W. W. Cheyne, Sir W. Church, Mr. J. Cantlie, Mr. L. V. Cargill, Canon Duckworth, Dr. J. F. Goodhart, Admiral Swinton C. Holland, Professor R. Tanner Hewlett, Lord Lochee, Sir M. Morris, Mr. Lawrie McGavin, Mr. L. A. Lawrence, Sir R. D. Powell, Colonel W. B. Leishman, R.A.M.C., Inspector-General James Porter, C.B. (D.G. Medical Department of the Navy), Lord Ridley, Mr. A. W. Mayo Robson, Dr. Guthrie Rankin, Sir Thomas Smith, Professor E. H. Starling, Dr. St. Clair Thomson, Dr. Frederick Taylor, Mr. William Turner, Colonel D. Wardrop, A.M.S., Colonel E. M. Wilson, Dr. Russell Wells, Canon Childe, Dr. F. de Havilland Hall, Dr. H. J. Wheeler, Dr. Douglas Reynolds, Dr. M. H. Spencer, Dr. R. J. Probyn-Williams.

### THE ADVERTISER'S A B C.

The Standard Advertisement Press Directory. 1909 Edition. Price 10s. 6d.

The twenty-third annual edition of this standard work, which has just been issued, is as usual handsomely produced. The contents include advertisement tariffs, circulations, fac-simile reproductions, and other particulars of the Press of the United Kingdom of a character peculiarly useful to advertisers. Comprehensive lists and serviceable information are also given of papers published abroad. The contents are conveniently arranged for speedy reference, and are carefully indexed. The volume is published by T. B. Browne, Ltd., 163 Queen Victoria Street, London.

